# 2-(2,2-Dimethyl-2,3-dihydro-1-benzofuran-7-yl­oxy)-*N*-(*o*-tol­yl)acetamide

**DOI:** 10.1107/S1600536810018672

**Published:** 2010-05-26

**Authors:** Wen-Sheng Li, Xian-Fu Luo, Yu Wang, Ai-Xi Hu

**Affiliations:** a College of Chemistry and Chemical Engineering, Hunan University, Changsha 410082, People’s Republic of China; b Hunan Research Institute of Chemical Industry, Changsha 410007, People’s Republic of China

## Abstract

In the title compound, C_19_H_21_NO_3_, the dihedral angle between the mean planes of the two benzene rings is 38.13 (12)°. The furan ring adopts an envelope-like conformation with the C atom bonded to the dimethyl groups displaced by 0.356 (2) Å from the plane through the other four atoms. In the crystal, mol­ecules are linked into inversion dimers by weak C—H⋯O inter­molecular inter­actions.

## Related literature

The title compound is a derivative of Carbofuran, a popular carbamate insecticide, see: Tomlin (1994 ▶). For related structures, see: Xu *et al.* (2005 ▶); Li *et al.* (2009 ▶). For bond-length data, see: Allen *et al.* (1987 ▶).
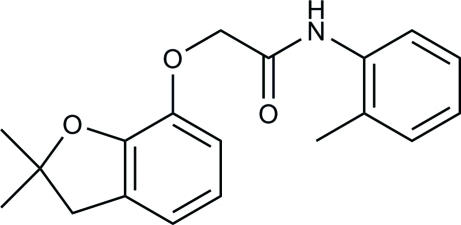


## Experimental

### 

#### Crystal data

C_19_H_21_NO_3_
*M* *_r_* = 311.37
Monoclinic, 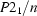

*a* = 9.0868 (18) Å
*b* = 8.9708 (18) Å
*c* = 20.230 (4) Å
β = 92.18 (3)°
*V* = 1647.9 (6) Å^3^
*Z* = 4
Mo *K*α radiation
μ = 0.09 mm^−1^
*T* = 293 K
0.32 × 0.28 × 0.21 mm

#### Data collection

Bruker APEXII area-detector diffractometer
Absorption correction: multi-scan (*SADABS*; Sheldrick, 1996 ▶) *T*_min_ = 0.985, *T*_max_ = 0.991
8246 measured reflections
2961 independent reflections
1649 reflections with *I* > 2σ(*I*)
*R*_int_ = 0.047

#### Refinement

*R*[*F* ^2^ > 2σ(*F* ^2^)] = 0.048
*wR*(*F* ^2^) = 0.130
*S* = 1.00
2961 reflections
211 parameters
H-atom parameters constrained
Δρ_max_ = 0.16 e Å^−3^
Δρ_min_ = −0.19 e Å^−3^

### 

Data collection: *APEX2* (Bruker, 2007 ▶); cell refinement: *SAINT* (Bruker, 2007 ▶); data reduction: *SAINT*; program(s) used to solve structure: *SHELXS97* (Sheldrick, 2008 ▶); program(s) used to refine structure: *SHELXL97* (Sheldrick, 2008 ▶); molecular graphics: *XP* in *SHELXTL* (Sheldrick, 2008 ▶); software used to prepare material for publication: *SHELXTL*.

## Supplementary Material

Crystal structure: contains datablocks I, global. DOI: 10.1107/S1600536810018672/jj2028sup1.cif

Structure factors: contains datablocks I. DOI: 10.1107/S1600536810018672/jj2028Isup2.hkl

Additional supplementary materials:  crystallographic information; 3D view; checkCIF report

## Figures and Tables

**Table 1:** Hydrogen-bond geometry (Å, °)

*D*—H⋯*A*	*D*—H	H⋯*A*	*D*⋯*A*	*D*—H⋯*A*
C12—H12*A*⋯O3^i^	0.97	2.60	3.561 (3)	174

Symmetry code: (i) 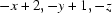.

**Fig. 1.**
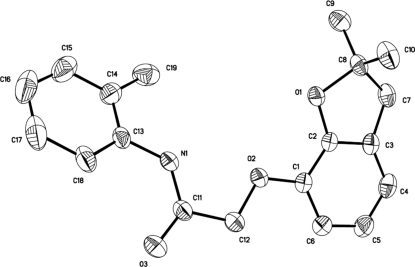
The structure of the title compound showing 30% probability displacement ellipsoids.

**Fig. 2.**
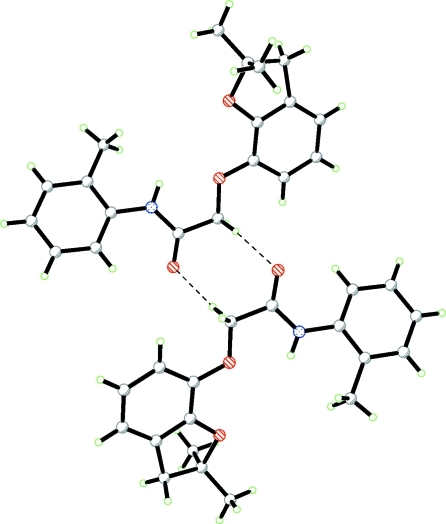
A packing diagram for the title compound. H atoms bonded to C atoms have been omitted for clarity. Dashed lines indicate weak C—H···O intermolecular interactions forminng dimers.

## supplementary crystallographic information

### Comment

The title compound, (I), C_19_H_21_NO_3_, is a derivative of the commercial compound Carbofuran (Tomlin, 1994), which is a popular carbamate insecticide. The dihedral angle between the mean planes of the two aromatic rings is 38.13 (12)° (Fig. 1). The five-membered furan ring adopts an envelope-like conformation.  All bond lengths (Allen *et al.* 1987) and angles are within normal ranges. The atom C1 deviates from the C1—C7/O1 plane with a distance of 0.356 (2) Å. Molecules are linked into dimers by weak C—H···O intermolecular interactions which helps stabilize crystal packing(Fig 2).

### Experimental

0.10 mol of 2,2-dimethy-2,3-dihydrobenzofuran-7-ol , 0.12 mol chloroacetic acid, 0.25 mol sodium hydrate and 70 ml distilled water were stirred and heated under reflux for 3 h.  The reaction mixture was then cooled to 283.15 K and 15 ml concentrated hydrochloric acid was added to give 2-(2,2-dimethyl-2,3-dihydrobenzofuran-7-yloxy)acetic acid as an amber solid of 21.91 g, yield 98.5%. Subsequently, 0.10 mol of dry 2-(2,2-dimethyl-2,3-dihydrobenzofuran-7-yloxy)acetic acid, 0.25 mol thionyl chloride and 80 ml anhydrous was stirred and heated at 353.15 K for 6h.  The excess thionyl chloride was then removed under reduced pressure.  The residue was cooled to 273.15 K, after which 0.10 mol *o*-toluidine and 0.20 mol triethylamine was added dropwise. After stirring for an additional 3 h, the reaction mixture was washed with water (3× 40 ml), and the excess toluene removed in vacuo. The residue was purified by recrystallization from a saturated ethanol solution, giving the title compound as a colourless crystalline solid. Single crystals suitable for X-ray diffraction were obtained by slow evaporation of an ethyl acetate solution at room temperature over a period of nine days. The identity of the title compound was confirmed by NMR and LC—MS spectroscopy.

### Refinement

Methyl H atoms were placed in calculated positions, with C–H = 0.96 Å, and torsion angles were refined, with *U*iso(H) = 1.5Ueq(C). Other H atoms were placed in geometrically idealized positions and refined as a riding model, with a N–H distance of 0.86 Å, C–H distances of 0.98Å (C3—H3), 0.93Å (aromatic H atoms) and 0.97Å (methylene H atoms). The constraint *U*iso(H) = 1.2Ueq(carrier) was applied.

### Crystal data

**Table:**

C_19_H_21_NO_3_	*F*(000) = 664
*M**_r_* = 311.37	*D*_x_ = 1.255 Mg m^−^^3^
Monoclinic, *P*2_1_/*n*	Melting point: 363.75 K
Hall symbol: -P 2yn	Mo *K*α radiation, λ = 0.71073 Å
*a* = 9.0868 (18) Å	Cell parameters from 3600 reflections
*b* = 8.9708 (18) Å	θ = 1.4–28°
*c* = 20.230 (4) Å	µ = 0.09 mm^−^^1^
β = 92.18 (3)°	*T* = 293 K
*V* = 1647.9 (6) Å^3^	Block, colourless
*Z* = 4	0.32 × 0.28 × 0.21 mm

### Data collection

**Table:**

Bruker APEXII area-detector diffractometer	2961 independent reflections
Radiation source: fine-focus sealed tube	1649 reflections with *I* > 2σ(*I*)
graphite	*R*_int_ = 0.047
φ and ω scan	θ_max_ = 25.2°, θ_min_ = 2.0°
Absorption correction: multi-scan (*SADABS*; Sheldrick, 1996)	*h* = −8→10
*T*_min_ = 0.985, *T*_max_ = 0.991	*k* = −10→9
8246 measured reflections	*l* = −19→24

### Refinement

**Table:**

Refinement on *F*^2^	Primary atom site location: structure-invariant direct methods
Least-squares matrix: full	Secondary atom site location: difference Fourier map
*R*[*F*^2^ > 2σ(*F*^2^)] = 0.048	Hydrogen site location: inferred from neighbouring sites
*wR*(*F*^2^) = 0.130	H-atom parameters constrained
*S* = 1.00	*w* = 1/[σ^2^(*F*_o_^2^) + (0.0561*P*)^2^] where *P* = (*F*_o_^2^ + 2*F*_c_^2^)/3
2961 reflections	(Δ/σ)_max_ = 0.001
211 parameters	Δρ_max_ = 0.16 e Å^−^^3^
0 restraints	Δρ_min_ = −0.19 e Å^−^^3^

### Special details

**Table:**

Experimental. ^1^H NMR in CDCl_3_ (300 MHz), delta: 1.49(s,6H,2CH_3_), 2.72(s,3H, ArCH_3_), 3.06(s,2H,CH_2_), 4.72 (s,2H, OCH_2_), 6.76~7.99(m,7H,C_6_H_3_,C_6_H_4_), 8.53(s,1H, NH). MS: 312.2(M+1)
Geometry. All esds (except the esd in the dihedral angle between two l.s. planes) are estimated using the full covariance matrix. The cell esds are taken into account individually in the estimation of esds in distances, angles and torsion angles; correlations between esds in cell parameters are only used when they are defined by crystal symmetry. An approximate (isotropic) treatment of cell esds is used for estimating esds involving l.s. planes.
Refinement. Refinement of *F*^2^ against ALL reflections. The weighted *R*-factor wR and goodness of fit *S* are based on *F*^2^, conventional *R*-factors *R* are based on *F*, with *F* set to zero for negative *F*^2^. The threshold expression of *F*^2^ > σ(*F*^2^) is used only for calculating *R*-factors(gt) etc. and is not relevant to the choice of reflections for refinement. *R*-factors based on *F*^2^ are statistically about twice as large as those based on *F*, and *R*- factors based on ALL data will be even larger.

### Fractional atomic coordinates and isotropic or equivalent isotropic displacement parameters (Å^2^)

**Table:**

	*x*	*y*	*z*	*U*_iso_*/*U*_eq_	
C7	0.4155 (3)	0.3064 (3)	0.23529 (11)	0.0590 (7)	
H7A	0.3140	0.2780	0.2254	0.071*	
H7B	0.4243	0.3430	0.2804	0.071*	
C1	0.6148 (2)	0.4224 (2)	0.09104 (10)	0.0478 (6)	
C2	0.5545 (2)	0.3488 (2)	0.14345 (10)	0.0452 (6)	
C3	0.4665 (2)	0.4205 (3)	0.18741 (10)	0.0508 (6)	
C6	0.5867 (3)	0.5737 (3)	0.08464 (11)	0.0625 (7)	
H6	0.6271	0.6269	0.0503	0.075*	
C8	0.5210 (3)	0.1749 (2)	0.22464 (10)	0.0534 (6)	
C5	0.4988 (3)	0.6465 (3)	0.12900 (12)	0.0697 (7)	
H5	0.4804	0.7479	0.1239	0.084*	
C4	0.4384 (3)	0.5705 (3)	0.18046 (12)	0.0661 (7)	
H4	0.3795	0.6198	0.2101	0.079*	
C10	0.6543 (3)	0.1807 (3)	0.27130 (12)	0.0741 (8)	
H10A	0.7220	0.1034	0.2601	0.111*	
H10B	0.6243	0.1664	0.3159	0.111*	
H10C	0.7014	0.2759	0.2677	0.111*	
C9	0.4475 (3)	0.0248 (3)	0.22421 (12)	0.0761 (8)	
H9A	0.3673	0.0243	0.1919	0.114*	
H9B	0.4107	0.0046	0.2672	0.114*	
H9C	0.5177	−0.0505	0.2133	0.114*	
O1	0.57451 (16)	0.20069 (15)	0.15680 (7)	0.0515 (4)	
O2	0.69720 (17)	0.34027 (15)	0.04836 (7)	0.0554 (5)	
N1	0.83327 (19)	0.16952 (19)	−0.03274 (8)	0.0500 (5)	
H1	0.7740	0.1490	−0.0019	0.060*	
C11	0.8592 (3)	0.3146 (3)	−0.04218 (11)	0.0540 (6)	
O3	0.9383 (2)	0.36591 (19)	−0.08343 (9)	0.0889 (7)	
C12	0.7796 (3)	0.4201 (2)	0.00151 (11)	0.0569 (6)	
H12A	0.8503	0.4844	0.0246	0.068*	
H12B	0.7138	0.4823	−0.0254	0.068*	
C13	0.8900 (3)	0.0448 (2)	−0.06653 (10)	0.0499 (6)	
C14	0.8094 (3)	−0.0865 (3)	−0.06628 (11)	0.0611 (7)	
C17	1.0776 (4)	−0.0718 (4)	−0.12804 (13)	0.0943 (11)	
H17	1.1673	−0.0671	−0.1486	0.113*	
C19	0.6654 (3)	−0.0952 (3)	−0.03299 (13)	0.0822 (8)	
H19A	0.6813	−0.0796	0.0137	0.123*	
H19B	0.6224	−0.1918	−0.0405	0.123*	
H19C	0.6001	−0.0199	−0.0508	0.123*	
C15	0.8674 (4)	−0.2095 (3)	−0.09801 (14)	0.0891 (10)	
H15	0.8155	−0.2989	−0.0985	0.107*	
C16	0.9987 (5)	−0.2025 (4)	−0.12855 (15)	0.1031 (13)	
H16	1.0347	−0.2863	−0.1497	0.124*	
C18	1.0232 (3)	0.0530 (3)	−0.09684 (10)	0.0664 (7)	
H18	1.0761	0.1417	−0.0963	0.080*	

### Atomic displacement parameters (Å^2^)

**Table:**

	*U*^11^	*U*^22^	*U*^33^	*U*^12^	*U*^13^	*U*^23^
C7	0.0495 (15)	0.0799 (17)	0.0482 (14)	0.0056 (13)	0.0113 (11)	−0.0089 (12)
C1	0.0495 (14)	0.0487 (14)	0.0452 (13)	0.0017 (11)	0.0031 (11)	−0.0074 (10)
C2	0.0489 (14)	0.0448 (13)	0.0419 (13)	0.0012 (11)	0.0030 (11)	−0.0075 (10)
C3	0.0457 (14)	0.0594 (15)	0.0475 (14)	0.0022 (12)	0.0034 (11)	−0.0117 (11)
C6	0.0803 (19)	0.0517 (15)	0.0558 (15)	0.0002 (13)	0.0053 (14)	−0.0017 (11)
C8	0.0530 (15)	0.0674 (15)	0.0406 (13)	0.0041 (12)	0.0144 (11)	0.0004 (11)
C5	0.089 (2)	0.0528 (14)	0.0672 (17)	0.0128 (14)	0.0046 (15)	−0.0085 (13)
C4	0.0663 (18)	0.0676 (17)	0.0647 (17)	0.0142 (14)	0.0053 (14)	−0.0208 (13)
C10	0.0674 (18)	0.103 (2)	0.0522 (16)	0.0144 (15)	0.0057 (14)	0.0012 (14)
C9	0.090 (2)	0.0737 (18)	0.0664 (17)	−0.0086 (16)	0.0263 (15)	0.0080 (14)
O1	0.0605 (10)	0.0524 (9)	0.0426 (9)	0.0055 (8)	0.0167 (7)	−0.0009 (7)
O2	0.0686 (11)	0.0503 (9)	0.0489 (9)	0.0012 (8)	0.0242 (8)	0.0000 (7)
N1	0.0551 (12)	0.0523 (12)	0.0438 (11)	−0.0020 (9)	0.0175 (9)	0.0037 (8)
C11	0.0602 (16)	0.0583 (16)	0.0444 (14)	−0.0096 (12)	0.0129 (12)	0.0007 (11)
O3	0.1140 (16)	0.0713 (12)	0.0856 (13)	−0.0173 (11)	0.0584 (12)	0.0004 (9)
C12	0.0635 (16)	0.0550 (14)	0.0536 (14)	−0.0072 (12)	0.0190 (12)	−0.0003 (11)
C13	0.0599 (16)	0.0579 (15)	0.0319 (12)	0.0128 (12)	0.0023 (11)	0.0017 (10)
C14	0.0787 (19)	0.0548 (15)	0.0485 (14)	0.0045 (14)	−0.0137 (13)	−0.0024 (12)
C17	0.106 (3)	0.128 (3)	0.0505 (17)	0.059 (2)	0.0147 (17)	0.0032 (18)
C19	0.088 (2)	0.0724 (18)	0.085 (2)	−0.0193 (16)	−0.0079 (17)	0.0098 (15)
C15	0.134 (3)	0.0651 (19)	0.0653 (19)	0.020 (2)	−0.029 (2)	−0.0111 (15)
C16	0.149 (4)	0.100 (3)	0.058 (2)	0.063 (3)	−0.014 (2)	−0.0204 (19)
C18	0.0742 (18)	0.0837 (18)	0.0421 (14)	0.0215 (15)	0.0121 (13)	0.0088 (13)

### Geometric parameters (Å, °)

**Table:**

C7—C3	1.495 (3)	C9—H9C	0.9600
C7—C8	1.540 (3)	O2—C12	1.423 (2)
C7—H7A	0.9700	N1—C11	1.337 (3)
C7—H7B	0.9700	N1—C13	1.418 (3)
C1—O2	1.378 (2)	N1—H1	0.8600
C1—C2	1.380 (3)	C11—O3	1.213 (2)
C1—C6	1.386 (3)	C11—C12	1.500 (3)
C2—O1	1.367 (2)	C12—H12A	0.9700
C2—C3	1.378 (3)	C12—H12B	0.9700
C3—C4	1.376 (3)	C13—C18	1.380 (3)
C6—C5	1.387 (3)	C13—C14	1.387 (3)
C6—H6	0.9300	C14—C15	1.390 (4)
C8—O1	1.492 (2)	C14—C19	1.497 (3)
C8—C9	1.503 (3)	C17—C16	1.374 (4)
C8—C10	1.508 (3)	C17—C18	1.386 (3)
C5—C4	1.376 (3)	C17—H17	0.9300
C5—H5	0.9300	C19—H19A	0.9600
C4—H4	0.9300	C19—H19B	0.9600
C10—H10A	0.9600	C19—H19C	0.9600
C10—H10B	0.9600	C15—C16	1.366 (4)
C10—H10C	0.9600	C15—H15	0.9300
C9—H9A	0.9600	C16—H16	0.9300
C9—H9B	0.9600	C18—H18	0.9300
			
C3—C7—C8	102.93 (17)	H9A—C9—H9C	109.5
C3—C7—H7A	111.2	H9B—C9—H9C	109.5
C8—C7—H7A	111.2	C2—O1—C8	106.68 (14)
C3—C7—H7B	111.2	C1—O2—C12	117.43 (16)
C8—C7—H7B	111.2	C11—N1—C13	129.02 (18)
H7A—C7—H7B	109.1	C11—N1—H1	115.5
O2—C1—C2	117.83 (18)	C13—N1—H1	115.5
O2—C1—C6	124.60 (19)	O3—C11—N1	125.5 (2)
C2—C1—C6	117.56 (19)	O3—C11—C12	118.5 (2)
O1—C2—C3	113.71 (19)	N1—C11—C12	116.00 (18)
O1—C2—C1	124.27 (18)	O2—C12—C11	110.67 (18)
C3—C2—C1	122.0 (2)	O2—C12—H12A	109.5
C4—C3—C2	120.0 (2)	C11—C12—H12A	109.5
C4—C3—C7	132.5 (2)	O2—C12—H12B	109.5
C2—C3—C7	107.51 (19)	C11—C12—H12B	109.5
C1—C6—C5	120.6 (2)	H12A—C12—H12B	108.1
C1—C6—H6	119.7	C18—C13—C14	121.3 (2)
C5—C6—H6	119.7	C18—C13—N1	120.9 (2)
O1—C8—C9	107.10 (17)	C14—C13—N1	117.8 (2)
O1—C8—C10	106.75 (18)	C13—C14—C15	117.6 (3)
C9—C8—C10	112.3 (2)	C13—C14—C19	121.1 (2)
O1—C8—C7	103.68 (16)	C15—C14—C19	121.3 (3)
C9—C8—C7	114.1 (2)	C16—C17—C18	119.9 (3)
C10—C8—C7	112.06 (19)	C16—C17—H17	120.1
C4—C5—C6	120.8 (2)	C18—C17—H17	120.1
C4—C5—H5	119.6	C14—C19—H19A	109.5
C6—C5—H5	119.6	C14—C19—H19B	109.5
C5—C4—C3	119.0 (2)	H19A—C19—H19B	109.5
C5—C4—H4	120.5	C14—C19—H19C	109.5
C3—C4—H4	120.5	H19A—C19—H19C	109.5
C8—C10—H10A	109.5	H19B—C19—H19C	109.5
C8—C10—H10B	109.5	C16—C15—C14	121.7 (3)
H10A—C10—H10B	109.5	C16—C15—H15	119.2
C8—C10—H10C	109.5	C14—C15—H15	119.2
H10A—C10—H10C	109.5	C15—C16—C17	120.0 (3)
H10B—C10—H10C	109.5	C15—C16—H16	120.0
C8—C9—H9A	109.5	C17—C16—H16	120.0
C8—C9—H9B	109.5	C13—C18—C17	119.6 (3)
H9A—C9—H9B	109.5	C13—C18—H18	120.2
C8—C9—H9C	109.5	C17—C18—H18	120.2

### Hydrogen-bond geometry (Å, °)

**Table:**

*D*—H···*A*	*D*—H	H···*A*	*D*···*A*	*D*—H···*A*
C12—H12A···O3^i^	0.97	2.60	3.561 (3)	174

Symmetry codes: (i) −*x*+2, −*y*+1, −*z*.
